# Chloroform-Methanol Residue of *Coxiella burnetii* Markedly Potentiated the Specific Immunoprotection Elicited by a Recombinant Protein Fragment rOmpB-4 Derived from Outer Membrane Protein B of *Rickettsia rickettsii* in C3H/HeN Mice

**DOI:** 10.1371/journal.pone.0124664

**Published:** 2015-04-24

**Authors:** Wenping Gong, Pengcheng Wang, Xiaolu Xiong, Jun Jiao, Xiaomei Yang, Bohai Wen

**Affiliations:** 1 State Key Laboratory of Pathogen and Biosecurity, Beijing Institute of Microbiology and Epidemiology, Fengtai, Beijing, China; 2 Department of Clinical Laboratory, the 105th Hospital of PLA, Hefei, Anhui, China; Texas A&M Health Science Center, UNITED STATES

## Abstract

The obligate intracellular bacteria, *Rickettsia rickettsii* and *Coxiella burnetii*, are the potential agents of bio-warfare/bio-terrorism. Here C3H/HeN mice were immunized with a recombinant protein fragment rOmp-4 derived from outer membrane protein B, a major protective antigen of *R*. *rickettsii*, combined with chloroform-methanol residue (CMR) extracted from phase I *C*. *burnetii* organisms, a safer Q fever vaccine. These immunized mice had significantly higher levels of IgG1 and IgG2a to rOmpB-4 and interferon-γ (IFN-γ) and tumor necrosis factor-α (TNF-α), two crucial cytokines in resisting intracellular bacterial infection, as well as significantly lower rickettsial loads and slighter pathological lesions in organs after challenge with *R*. *rickettsii*, compared with mice immunized with rOmpB-4 or CMR alone. Additionally, after challenge with *C*. *burnetii*, the coxiella loads in the organs of these mice were significantly lower than those of mice immunized with rOmpB-4 alone. Our results prove that CMR could markedly potentiate enhance the rOmpB-4-specific immunoprotection by promoting specific and non-specific immunoresponses and the immunization with the protective antigen of *R*. *rickettsii* combined with CMR of *C*. *burnetii* could confer effective protection against infection of *R*. *rickettsii* or *C*. *burnetii*.

## Introduction

Rocky Mountain spotted fever (RMSF) is a serious and potentially life-threatening infectious disease, which is caused by *Rickettsia rickettsii*, an obligate intracellular Gram-negative bacterium naturally transmitted by tick bites [1]. Initial signs and symptoms of RMSF include sudden onset of fever, headache, and muscle pain, as well as a history of tick bit or contact, followed by development of rash [2,3]. The seriously infected patients will develop signs and symptoms of acute lung edema, renal failure, or encephalitis [2,3], which may be fatal, due to wide spread vasculitis caused by rickettsial infection of endothelial cells lining small blood vessels in the vital organs [4,5].

*Coxiella burnetii*, a rickettsia-like bacterium belonging to order *Legionellales*, is the etiological agent of Q fever in humans. Human Q fever is generally acquired via the respiratory route by inhalation of infectious aerosols produced by domestic livestock [6] such as sheep or goats [7,8]. Human Q fever presents a flu-like syndrome and may develop pneumonia in serious *C*. *burnetii* infection [9,10]. Acute Q fever may progress to chronic disease complicated by endocarditis, chronic hepatitis, and/or osteomyelitis [7,11].

Both *R*. *rickettsii* and *C*. *burnetii* are recognized as potential agents of bio-warfare/bio-terrorism due to their production and release of lyophilized particles through aerosol, which seems to be particularly urgent to develop effective vaccines against them. Early attempts to develop vaccines against RMSF or Q fever focused on classical approaches for preparation of an inactivated whole cell vaccine (WCV), including propagation of organisms in animals or cells, purification of the organisms from infected tissues or cells, and inactivation of the purified organisms. However, the inactivated *R*. *rickettsii* organisms has been shown to reduce mortality rates but have failed to prevent disease onset [12,13].

WCV against Q fever is usually prepared with organisms isolated from the embryonated eggs infected with phase I *C*. *burnetii*, which is effective in protecting human and animals from *C*. *burnetii* infection [14,15]. Whereas the use of this WCV was frequently accompanied by adverse reactions, such as sterile abscesses and granulomas at the inoculation site in humans previously sensitized by natural infection of *C*. *burnetii*, which limit its use in humans [16]. A novel type of Q fever vaccine was developed by extracting *C*. *burnetii* organisms with chloroform-methanol, and the chloroform-methanol residue (CMR) is an efficacious alternative to WCV with less adverse reactions [17]. Furthermore, a complex nutrient medium that supported a substantial cell-free growth of *C*. *burnetii* was developed [18] and the axenic culture of *C*. *burnetii* lays a critical foundation for easily producing CMR vaccine on a large scale.

Previous studies have revealed that animals treated with inactivated phase I *C*. *burnetii* organisms had a significant increase in resistance to tumors, virus, bacteria or protozoans by the specific and nonspecific immunity modulated by the organisms, indicating that phase I *C*. *burnetii* is a potent immunopotentiator [19–21]. CMR of *C*. *burnetii* can induce nonspecific immunoresponses, producing high levels of interferon-γ (IFN-γ) and tumor necrosis factor-α (TNF-α) in hosts [22,23], which inhibit viral, protozoan and bacterial infections via activation of bactericidal systems of macrophages and cytotoxicity of NK cells [24]. Furthermore, CMR of *C*. *burnetii* can increase production of macrophage-derived cytokines such as GM-CSF and IL-1 to activate dendritic cells and it also can increase production of lymphokines and expression of Ia MHC class II antigen of lymphocytes, leading to enhanced antigen processing and potentiation of antigen-specific humoral and cellular immunoresponses in hosts [23]. Outer membrane B (OmpB), a major surface protein of rickettsiae, has been well demonstrated to be an important protective antigen [25] as well as a crucial virulent factor of rickettsiae [26–28].

In this study, the whole gene (4965 bp) encoding OmpB of *R*. *rickettsii* were divided into 5 fragments to express in prokaryotic cells, resulting in 5 recombinant proteins (rOmpB-1 to 5). Following the analysis of immunoprotective efficacy, rOmpB-4 was proved to be the best one to confer protection against *R*. *rickettsii* infection in mice. And thus rOmpB-4 mixed with *C*. *burnetii* CMR was applied to immunize mice. Our results revealed that CMR could potentiate the rOmpB-4-specific immunoprotection to effectively resist *R*. *rickettsii* infection as well as elicit CMR-specific protection to counter *C*. *burnetii* infection in mice. Furthermore, the potential mechanism of the efficient immunoprotections conferred by the combination of rOmpB-4 and CMR was also investigated.

## Materials and Methods

### Bacterial strains

*Rickettsia rickettsii* (Sheila Smith strain) were cultured in Vero cells and isolated by isopycnic density gradient centrifugation as per conventional methods [29]. The number of *R*. *rickettsii* or viable rickettsial organisms in suspension was detected by quantitative polymerase chain reaction (qPCR) specific for *R*. *rickettsii* [30] or plaque assay [31]. *Coxiella burnetii* (Xinqiao strain, phase I) was grown in the acidified citrate cysteine medium (ACCM) as described previously [18]. The purified *C*. *burnetii* organisms were inactivated with formalin and extracted 2 times with chloroform-methanol (4:1) to obtain CMR fraction according to the procedures described previously [23]. The purified organisms were inactivated with formalin as whole cell antigens (WCA).

### Mice

Male C3H/HeN mice at 6–7 weeks old were purchased from Vital River Laboratories (Beijing, China). All animal experiments were carried out according to the guidelines of authors' institution. The protocol was approved by the Institute of Animal Care and Use Committee (IACUC No: AMMS-2014-020) at Academy of Military Medical Sciences (AMMS) and all efforts were made to minimize mice suffering.

### Preparation of recombinant proteins

**Fig 1:**
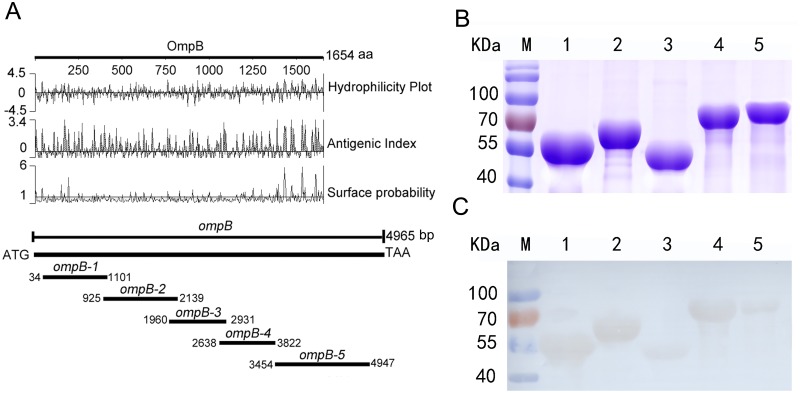
Diagram of preparing recombinant OmpB fragments (rOmpBs). The full-length sequence of *ompB* was divided into 5 fragments (named as *ompB-1* to *-5*) according to hydrophilicity, antigenic index, and surface probability (A). Five recombinant OmpB fragments (named as rOmpB-1 to -5) purified from *E*. *coli* cell lysate were separated by 10% SDS-PAGE and stained by G-250 Coomassie Brilliant Blue (B) and immunoblotted with sera from mice infected with *R*. *rickettsii* (C): lane M, protein molecular mass markers; lanes 1 to 5, rOmpB-1 to -5 (C).

The open reading frames (ORFs) of *ompB* (4965 bp, ABV76666.1) of *R*. *rickettsii* were divided into 5 fragments (named as *ompB-1* to *ompB-5*) according to hydrophilicity, antigenic index, and surface probability (Fig 1A). Five *ompB* fragments were amplified by polymerase chain reaction (PCR) from genomic DNA of *R*. *rickettsii* (GenBank accession number: CP000848) with cognate primer pairs (S1 Table), respectively. Each of 5 *ompB* fragments was inserted into pET32a (+) plasmid (Novagen, Madison, WI) to construct a recombinant plasmid that was used to transform *Escherichia coli* BL21 cells (Novagen, Madison, WI) according to conventional procedures [32]. The expressed recombinant OmpB fragments (rOmpB-1 to 5, rOmpBs) were respectively purified from the cognate gene-transformed *E*. *coli* cells using Ni-NTA affinity resin (Qiagen GmbH, Hilden, Germany) as per the manufacture’s instruction and the purified rOmpBs were subjected to 10% SDS-PAGE and immunoblotted with sera from mice infected with *R*. *rickettsii* following the methods described previously [33]. The endotoxin of the purified recombinant proteins were removed with Toxin Eraser (GenScript, Piscataway, NJ) [34].

### Evaluation of protective efficacy of rOmpBs

C3H/HeN mice (*n* = 5) were immunized with the 5 rOmpBs, respectively. Briefly, each mouse was injected subcutaneously (i.s.) with 30 μg of each rOmpB in 200 μl PBS mixed with complete Freund's adjuvant (CFA, Sigma-Aldrich, MO). Then, 20 μg of cognate rOmpB in 200 μl PBS mixed with incomplete FA (IFA, Sigma-Aldrich, MO) were injected intraperitoneally (i.p.) on day 28 and 42 after primary immunization. In parallel, WCA of *R*. *rickettsii* and PBS alone were used to immune mice at same doses and procedures described above as positive and negative controls, respectively. Fourteen days after last immunization, each mouse was challenged i.p. with a sublethal dose of viable *R*. *rickettsii* (6 × 10^6^ PFU). On day 5 after the challenge, each mouse was sacrificed to determine rickettsial loads in spleen, liver, and lung by qPCR described previously [35].

### Mouse immunization with rOmpB-4 and CMR

Each mouse per group (*n* = 5 mice) was injected i.s. with 30μg of rOmpB-4 and 30μg of CMR in 200μl PBS (rOmpB-4-CMR group), with 30μg of rOmpB-4 in 200μl PBS (rOmpB-4 group), or with 30μg of CMR in 200μl PBS (CMR group). Fourteen days after the primary immunization, each mouse was injected i.p. with 20μg of rOmpB-4 and 30μg of CMR in 200μl PBS (rOmpB-4-CMR group), with 20μg of rOmpB-4 in 200μl PBS (rOmpB-4 group), or with 30μg of CMR in 200μl PBS (CMR group). Fourteen days later, each mouse was challenged i.p. with a sublethal dose of *R*. *rickettsii* (6 × 10^6^ PFU). On day 5 past challenge, mice were sacrificed and their livers, spleens, and lungs were harvested for determination of *R*. *rickettsii* by qPCR [35].

In addition, other 2 groups of mice (*n* = 5) were immunized and boosted with rOmpB-4 mixed with CMR (rOmpB-4-CMR group) and mixed with PBS (rOmpB-4 group) at the same doses and procedures described above, respectively. Fourteen days after final immunization, each mouse was challenged i.p. with sublethal dose of *C*. *burnetii* (1 × 10^7^ PFU). Five days later, mice were sacrificed and their livers, spleens, and lungs were harvested for determination of *C*. *burnetii* by qPCR [36] Additionally, the spleen weight of mouse in *R*. *rickettsii* or *C*. *burnetii* infection groups was also determined.

### Histopathological analysis

A part of spleen, liver, or lung from each sacrificed mouse per group was collected for histopathological examination. The tissue samples were fixed in 4% (vol/vol) formaldehyde overnight, embedded in paraffin, sectioned at 5-μm thickness, and stained by hematoxylin and eosin for evaluation of histopathology under an Olympus DP71 microscope.

### Determination of specific antibodies in mouse sera

Blood samples were collected from the tail veins of mice per immunized group and pooled together on day 7, 14, 21, and 28 after primary immunization, respectively. Anti-rOmpB-4 IgGs were detected by enzyme-linked immunosorbent assay (ELISA). Briefly, 96-well plate (Nunc, Shanghai, China) was coated with 1.5 μg/ml rOmpB-4 overnight and incubated with mouse sera at the dilution of 1:1000. Then, the IgG, IgG1, or IgG2a to rOmpB-4 was determined with goat anti-mouse IgG, IgG1, or IgG2a HRP-conjugated antibodies (1:5000) and a TMB substrate kit (eBioscience, San Diego, CA) according to previous methods [32]. Absorbance at 450nm was analysed with a UVM 340 microplate reader (Asys Hitech GmbH, Eugendorf, Austria). Anti-*C*. *burnetii* phase I/II IgGs were detected by indirect immunofluorescence assay (IFA) as per methods described previously [37]. The phase I or II *C*. *burnetii*-coated slide was incubated with sera from mice immunized with rOmpB-4 mixed with CMR at two-fold dilution (initial at the dilution of 1:100) in PBS for 45 min at 37°C. After three washes with PBS, the *C*. *burnetii* cells on the slides were incubated with a 1:100 dilution of FITC-conjugated goat anti-mouse IgGs (eBioscience, San Diego, CA) for 45 min at 37°C. After another three washes, the coxiella cells on the slides were observed under a fluorescence microscope (Olympus BX60).

### Serum neutralization assay of *R*. *rickettsii*

The human endothelial hybrid cell line (EA.hy 926, ATCC), the host cells of *R*. *rickettsii*, were cultured in DMEM containing 15% heat-inactivated FBS. The pooled sera collected from rOmpB-4-CMR group, rOmpB-4 group, or CMR group mice on day 28 after primary immunization were inactivated at 56°C for 30 min and filter sterilized [38]. And then 150 μl of each serum sample was mixed with *R*. *rickettsii* cells in 150 μl of DMEM (3 ×10^7^ PFU/ml) at room temperature for 60 min. After which the serum-rickettsial mixture was added to 3 × 10^5^ host cells in 2.7 ml of DMEM containing 1% heat-inactivated FBS. This mixture was divided into 3 replicate wells in a 24-well plate (Corning, Corning, NY) and cultured at 37°C for 4 h [38]. After 3 times washing, the remaining cells in each well were collected for DNA extraction with DNeasy Blood & Tissue Kit (Qiagen GmbH, Hilden, Germany). The DNA samples were evaluated by qPCR with primers specific for *R*. *rickettsii* [35].

### Cytokine determination

The blood samples were collected from the tail vein of rOmpB-4-CMR group, rOmpB-4 group, or CMR group mice and pooled together to obtain a serum sample on days 7, 14, or 21 after primary immunization. And IFN-γ and TNF-α in the serum sample were determined using a Luminex Bio-Plex 200 IS 100 instrument (BIO-RAD, Hercules, CA) with multiplex kits and related reagents produced by Affymetrix (Santa Clara, CA).

### Statistical analysis

All statistics were computed using SAS statistical software (version 9.1, SAS Institute Inc., Cary, NC). The statistical significances of the differences in protective efficacy among rOmpBs were assayed using variance (ANOVA) procedure or Kruskal-Wallis test (NPAR1WAY Procedure) according to their normality and homogeneity of variances, followed by between-group comparison with Student-Newman-Keuls Test. The differences in protective efficacy among groups after challenge with *R*. *rickettsii* or *C*. *burnetii* and the differences in serum neutralization and ELISA or cytokines were assayed using Student’s *t-*test or Wilcoxon Two-Sample test, and *P*<0.05 was considered significantly different.

## Results

### Evaluation of protective efficacy of rOmpBs

The recombinant OmpBs, rOmpB-1 (57 KDa), rOmpB-2 (62 KDa), rOmpB-3 (53 KDa), rOmpB-4 (61 KDa), and rOmpB-5 (72 KDa), were expressed as His_6_-tagged fusions in *E*. *coli* cells and purified using Ni-NTA affinity resin under denaturing conditions. The purified rOmpBs were separated by SDS-PAGE (Fig 1B) and immunoblotted with sera from mice experimentally infected with *R*. *rickettsii* (Fig 1C). All of the 5 OmpBs were recognized by the sera, and rOmpB-1, rOmpB-2, or rOmpB-4 reacted more strongly with the sera compared to rOmpB-3 or rOmpB-5 (Fig 1C).

**Fig 2:**
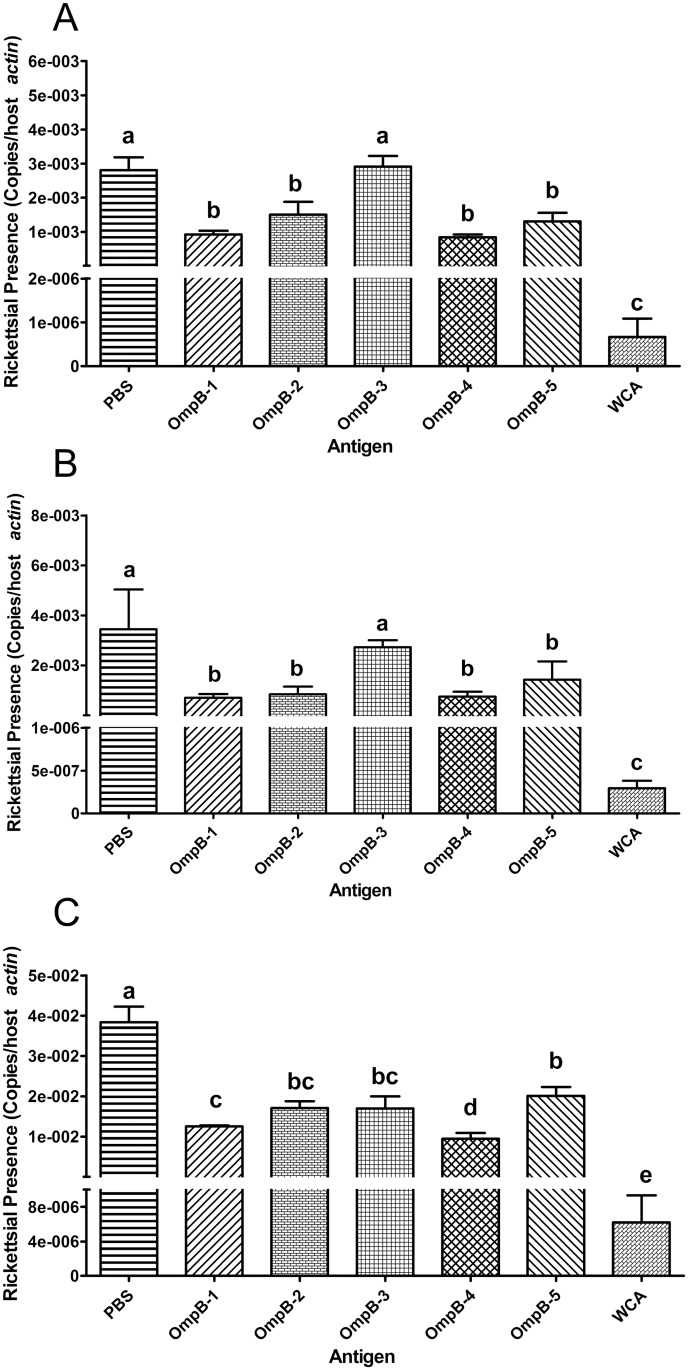
Evaluation of immunoprotective efficacy of rOmpBs. C3H/HeN mice were immunized with each of the rOmpBs, WCA, or PBS, followed by a sublethal challenge with *R*. *rickettsii*. Five days after challenge, the mice were sacrificed and the rickettsial load in their livers (A), spleens (B), or lungs (C) was determined using *R*. *rickettsii*-specific qPCR. The rickettsial load is expressed as the ratio of *R*. *rickettsii ompB* to murine *actin* gene copies (S2 Table), and the results were analyzed by variance (ANOVA) procedure or Kruskal-Wallis test (NPAR1WAY Procedure) according to their normality and homogeneity of variances, followed by between-group comparison with Student-Newman-Keuls Test. Data were presented as mean + SEM. (*n* = 5). Means with different letters are significantly different (*P*< 0.05).

The 5 rOmpBs were used to immunize C3H/HeN mice, respectively. After challenge with *R*. *rickettsii*, the rickettsial load in livers or spleens of mice immunized with rOmpB-1, rOmpB-2, rOmpB-4, rOmpB-5, or WCA of *R*. *rickettsii* and that in lungs of mice immunized with any of the 5 rOmpBs or WCA of *R*. *rickettsii* were significantly lower compared with mice mock-immunized with PBS. And mice immunized with rOmpB-4 consistently harbored lower levels of *R*. *rickettsii* in these organs compared with mice immunized with any other rOmpB (Fig 2A–2C).

### Immunoprotection induced by rOmpB-4 and/or CMR

**Fig 3:**
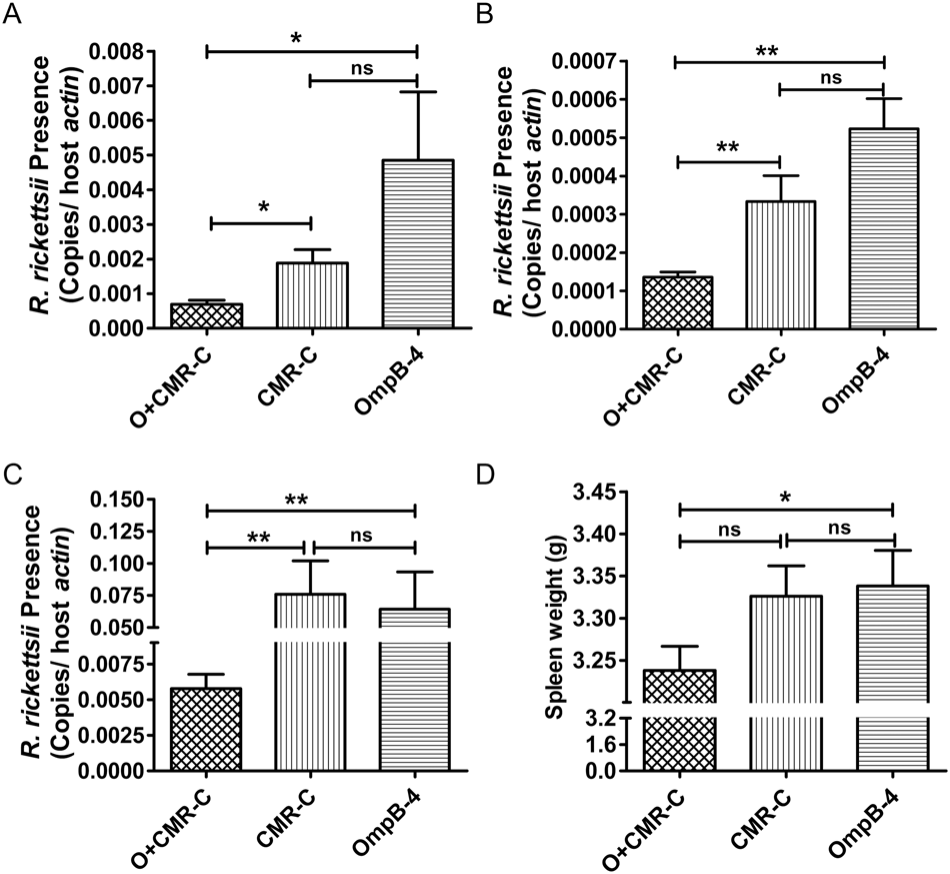
Comparison of immunoprotective efficacy between combination group and individual group. C3H/HeN mice were immunized with rOmpB-4 combined with *C*. *burnetii* CMR (O+CMR-C), *C*. *burnetii* CMR alone (CMR-C), or rOmpB-4 alone (rOmpB-4). Day 14 after the last immunization, mice were challenged with *R*. *rickettsii*, and mice were sacrificed and their livers (A), spleens (B) and lungs (C) were collected day 5 post challenge. In *R*. *rickettsii*-specific qPCR assay, the data was expressed as the ratio of *R*. *rickettsii ompB* to murine *actin* gene copies (S2 Table). The spleen weight of mice in each group was also been determined (D). The significant difference between two groups was compared with the Student’s *t-*test or Wilcoxon two-sample test according to their normality and homogeneity of variance. All data were presented as mean + SEM. (*n* = 5). *P*<0.05 was considered significantly different. *, *P*<0.05; **, *P*<0.01; ns, no significance.

To evaluate the potentiation of *C*. *burnetii* CMR, mice immunized with rOmpB-4 combined with CMR, or rOmpB-4/CMR alone were challenged with *R*. *rickettsii*. As a result, the rickettsial load in livers (*P* < 0.05, Fig 3A), spleens (*P* < 0.01, Fig 3B), or lungs (*P* < 0.01, Fig 3C) of mice immunized with rOmpB-4 combined with CMR was significantly lower than that with rOmpB-4 or CMR alone. The rickettsial load in livers or spleens of mice immunized with CMR alone were lower than that in cognate organs of mice immunized with rOmpB-4 alone, but which was not significantly different (*P* > 0.05, Fig 3A and 3B). The spleen weight of mice immunized with rOmpB-4 combined with CMR was significantly lighter than that of mice immunized with rOmpB-4 alone (*P* < 0.05, Fig 3D).

**Fig 4:**
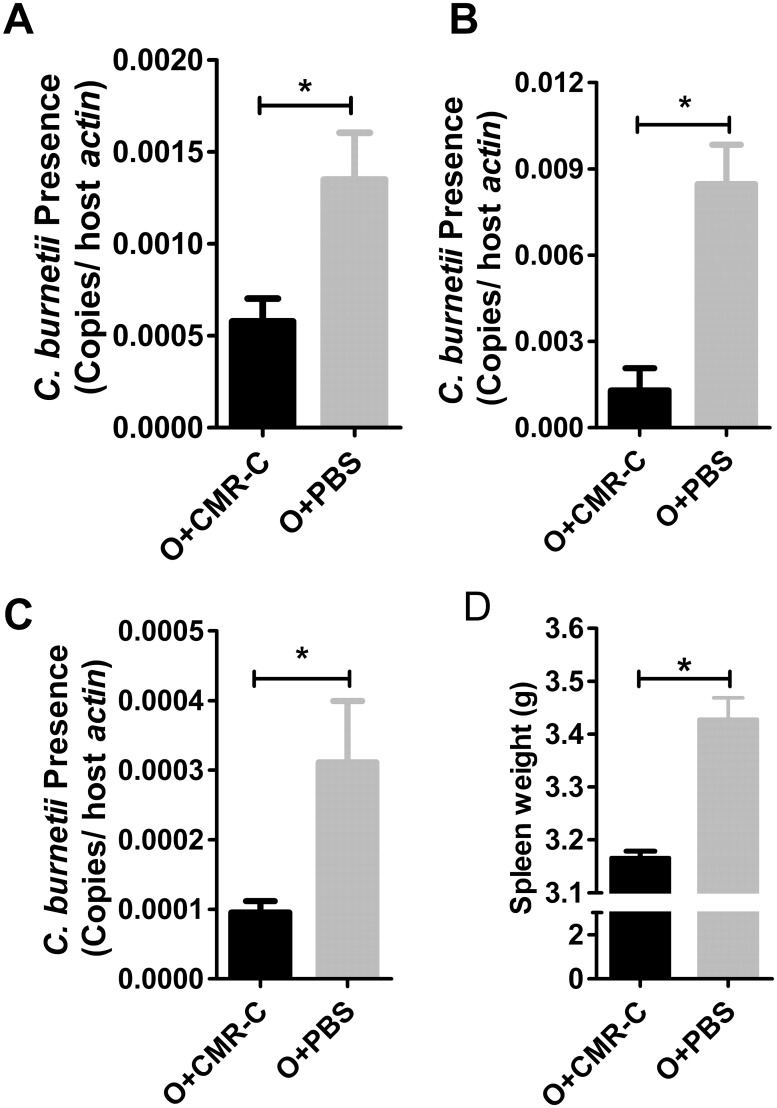
Comparison of protective efficacy between rOmpB-4 combined with *C*. *burnetii* CMR and rOmpB-4 alone. C3H/HeN mice were immunized with rOmpB-4 combined with *C*. *burnetii* CMR (O+CMR-C) or PBS (O+PBS). Day 14 after the last immunization, mice were challenged with *C*. *burnetii*, and mice were sacrificed and their livers (A), spleens (B) and lungs (C) were collected day 5 post challenge. In *C*. *burnetii*-specific qPCR assay, the data was expressed as the ratio of *C*. *burnetii 23S rRNA* to murine *actin* gene copies (S2 Table). The spleen weight of mice in each group was also been determined (D). The significant difference between two groups was compared with the Student’s *t-*test or Wilcoxon two-sample test according to their normality and homogeneity of variance. Data were presented as mean + SEM. (*n* = 5). *P*<0.05 was considered significantly different. *, *P*<0.05.

Additionally, mice immunized with rOmpB-4 combined with *C*. *burnetii* CMR or rOmpB-4 alone were challenged with *C*. *burnetii*, after which the coxiella load in livers, spleens or lungs of mice immunized with rOmpB-4 combined with CMR was significantly lower than that of mice immunized with rOmpB-4 alone (*P*<0.05, Fig 4A–4C), and the spleen weight of mice immunized with rOmpB-4 combined with CMR was significantly lighter than that of mice immunized with rOmpB-4 alone (*P* < 0.05, Fig 4D).

### Histopathological examination

**Fig 5:**
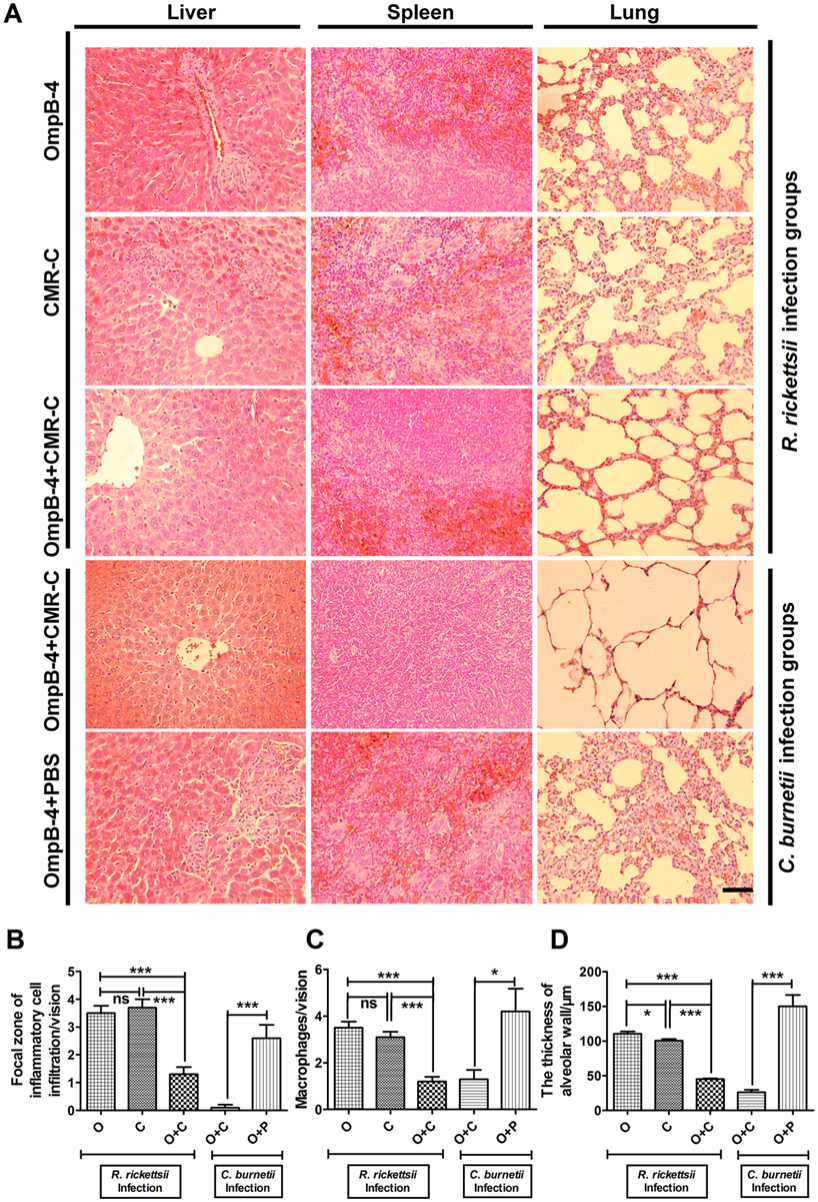
Pathological lesions after *R*. *rickettsii* or *C*. *burnetii* challenge. Liver, spleen, and lung tissues were collected from mice infected with *R*. *rickettsii* or *C*. *burnetii* for pathological examination (A, original magnifications 400, bar = 200μm), respectively. The focal zone of inflammatory infiltrates in livers (B), the number of macrophage number in spleens (C), and the mean thickness of alveolar wall in lungs (D) of mice were observed. The lesions in liver, spleen, or lung were quantified (*n* = 10 lesion high powered fields), and the differences between the groups were compared using Student’s *t-*test or Wilcoxon two-sample test according to their normality and homogeneity of variance. *, *P*<0.05; ***, *P*<0.001; ns, no significance.

As shown in Fig 5A, lobular hepatitis, inflammatory cell infiltration, macrophagocytes, and interstitial pneumonia were observed in liver, spleen, and lung from mice infected by *R*. *rickettsii* or *C*. *burnetii*, respectively. After challenge with *R*. *rickettsii*, the focal zone of inflammatory infiltrates in livers (Fig 5B), the number of macrophage number in spleens (Fig 5C), and the mean thickness of alveolar wall in lungs (Fig 5D) of mice immunized by rOmpB-4 combined with CMR-C were significantly less or thinner compared with those of mice immunized by either rOmpB-4 or CMR-C. Meanwhile, after challenge with *C*. *burnetii*, these pathological alters in livers, spleens, and in lungs of mice immunized by rOmpB-4 combined with CMR-C were also significantly slighter than those of mice immunized by rOmpB-4 alone (Fig 5A–5D).

### Specific antibodies in sera from mice immunized with rOmpB-4 and/or CMR

**Fig 6:**
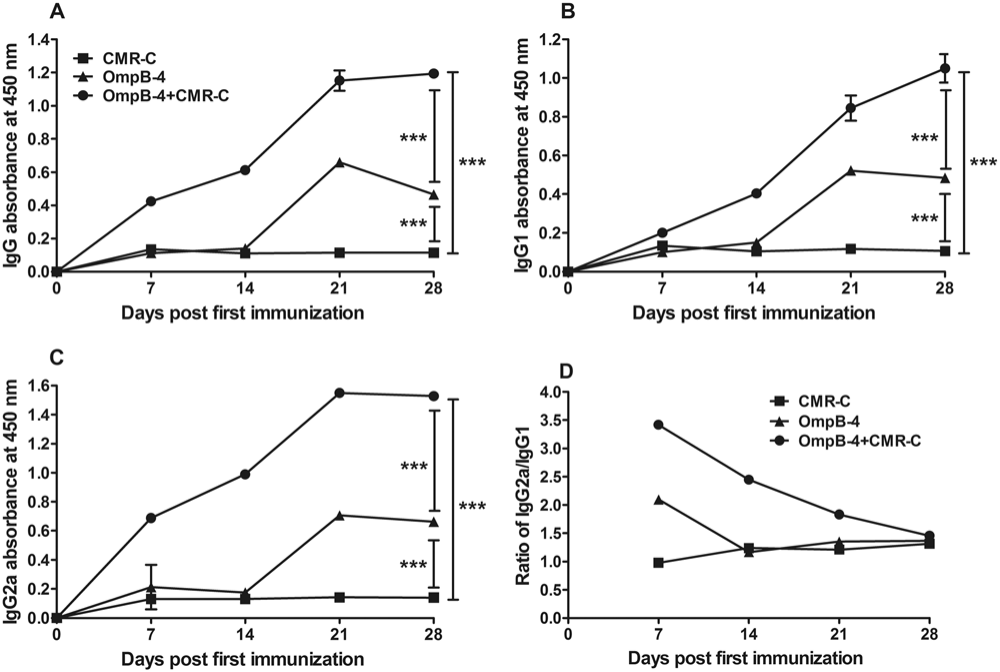
Specific antibodies determined by ELISA. Sera samples were collected from mice immunized with rOmpB-4 combined with *C*. *burnetii* CMR (O+CMR-C), *C*. *burnetii* CMR alone (CMR-C), or rOmpB-4 alone (rOmpB-4) on days 7, 14, 21, and 28 days after first immunization, respectively. IgG (A), IgG1 (B), or IgG2a (C) to rOmpB-4 in sera was determined by ELISA and the ratio of IgG2a/IgG1 in each serum sample was also compared (D). The statistically significant differences of OD_450_ on 28 days after first immunization among groups were analyzed using the Student’s *t-*test or Wilcoxon two-sample test according to their normality and homogeneity of variance. Results were expressed as mean ± SD (*n* = 3). *P*<0.05 was considered significantly different. ***, *P*<0.001.

The specific antibodies (IgG, IgG1, and IgG2a) to rOmpB-4 in sera collected from mice immunized with rOmpB-4 combined with *C*. *burnetii* CMR or rOmpB-4/CMR alone on days 7, 14, 21, and 28 after primary immunization were determined by ELISA. Mice immunized with rOmpB-4 combined with CMR produced a significantly higher level of IgG (Fig 6A, *P*<0.001), IgG1 (Fig 6B, *P*<0.001) or IgG2a (Fig 6C, *P*<0.001) compared mice immunized with rOmpB-4 or CMR alone on days 7, 14, 21, and 28 after primary immunization. Additionally, the ratio of IgG2a/IgG1 to rOmpB-4 of mice immunized with rOmpB-4 combined with CMR was markedly higher than that of mice immunized with rOmpB-4 or CMR alone on days 7, 14, and 21 after primary immunization (Fig 6D).

**Fig 7:**
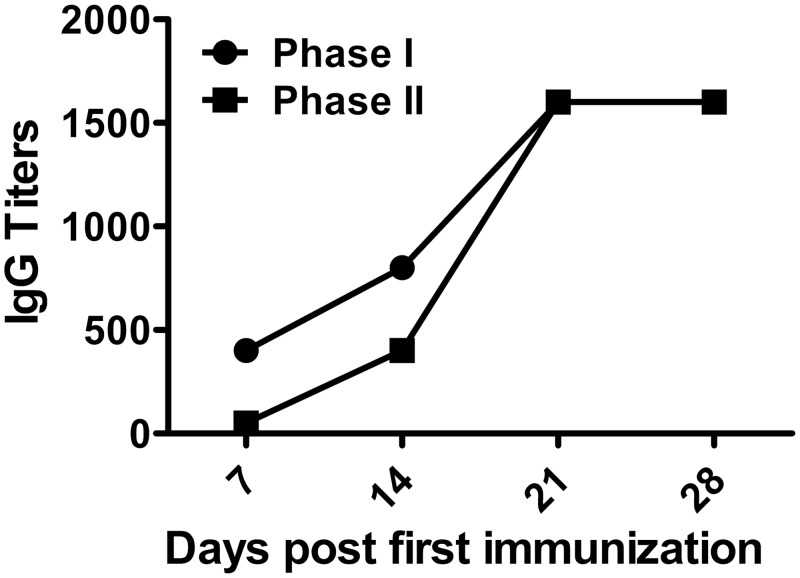
Specific antibodies determined by IFA. Serum samples were collected from mice immunized with rOmpB-4 combined *C*. *burnetii* CMR on days 7, 14, 21, and 28 after first immunization, respectively. Anti-*C*. *burnetii* phase I/II IgG titers was evaluated by IFA.

The IgGs to *C*. *burnetii* phase I or Phase II antigen were detected by IFA. The results displayed that both anti-phase I and Phase II IgGs were dramatically rising till day 21 after primary immunization and then keeping at the high level on day 28 (Fig 7).

### Neutralization of rickettsiae with sera from mice immunized with rOmpB-4 and/or CMR

**Fig 8:**
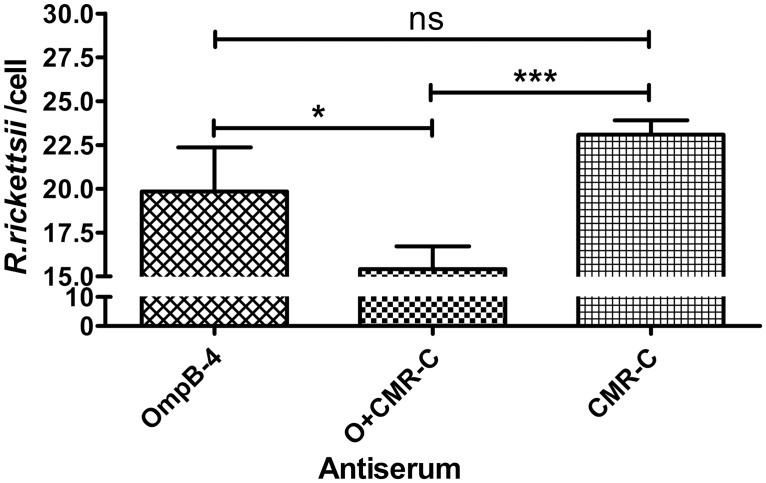
Neutralization of *R*. *rickettsii* by sera. Viable *R*. *rickettsii* were incubated with sera from mice immunized with rOmpB-4 combined with *C*. *burnetii* CMR (O+CMR-C), rOmpB-4 alone (rOmpB-4), or *C*. *burnetii* CMR alone (CMR-C). Sixty minutes later, the mixture of rickettsiae and each serum sample was added into EA.hy 926 cells for a 4-hour incubation. After which the number of *R*. *rickettsii* in host cells was determined by *R*. *rickettsii*-specific qPCR. Values are presented as the mean with standard deviations. The statistically significant differences among groups were analyzed using the Student’s *t-*test or Wilcoxon Two-Sample Test based on their normality and equality of variances and are indicated as follows: *, *P*<0.05; ***, *P*<0.001; ns, no significance. All data were presented as mean + SD (*n* = 3).

As Fig 8 showed, the total amount of rickettsiae treated with sera from mice immunized with rOmpB-4 combined with *C*. *burnetii* CMR was significantly lower than that treated with sera from mice with rOmpB-4 alone (*P*<0.05) or CMR alone (*P*<0.001), while that treated with sera from mice immunized with rOmpB-4 alone was lower, but not significantly, than that treated with sera from mice immunized with CMR alone (*P>*0.05).

### Cytokines in sera from mice immunized with rOmpB-4 and/or CMR

**Fig 9:**
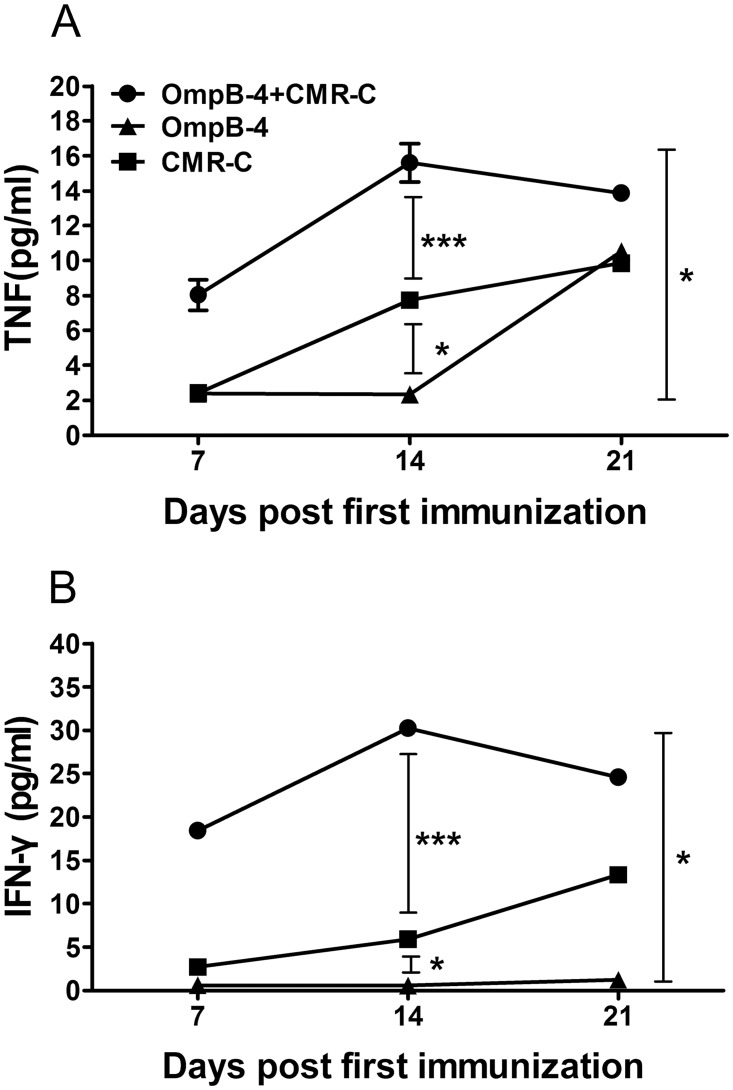
Detection of TNF-α and IFN-γ in sera. The pooled sera collected from mice immunized with rOmpB-4 combined with *C*. *burnetii* CMR (O+CMR-C), *C*. *burnetii* CMR alone (CMR-C), or rOmpB-4 alone (rOmpB-4) on days 7, 14, and 21 after first immunization, respectively. TNF-α (A) or IFN-γ (B) in the sera was detected with a Multiplex MouseTh1/Th2 cytokine kit. The statistically significant differences in TNF-α or IFN-γ levels among groups on 14 days after primary immunization were analyzed using the Student’s *t-*test or Wilcoxon two-sample test according to their normality and homogeneity of variance. Results were expressed as mean ± SD (*n* = 3). *P*<0.05 was considered significantly different. *, *P*<0.05; ***, *P*<0.001.

As shown in Fig 9, the content of TNF-α or IFN-γ in sera from mice immunized with rOmpB-4 combined with *C*. *burnetii* CMR was significantly higher than those in sera from mice immunized with rOmpB-4 (*P*<0.05) or CMR (*P*<0.001) alone through the immunization process, especially day 14 after primary immunization.

## Discussion

*R*. *rickettsii* and *C*. *burnetii* are both the obligate intracellular pathogens parasiting respectively in vascular endothelial cells and monocytes/macrophages. OmpB, an important virulence factor of rickettsiae [28,39], is the most abundant and high-molecular-mass surface protein of rickettsiae [40]. OmpB has been proved to be a good protective antigen capable of eliciting specific protective immunoresponses in animal models by eliciting the production of specific antibodies to inhibit rickettsial adhesion and invasion of host cells as well as opsonize macrophages or activate complements to kill rickettsiae in hosts [40], making it good candidate for subunit vaccine against rickettsial infection [39]. Here *ompB* gene of *R*. *rickettsii* was divided into 5 fragments to result in 5 recombinant proteins and our results showed that any of the rOmpBs except rOmpB-3 could offer a significant protection against *R*. *rickettsii* infection and rOmpB-4 was the best one among the protective rOmpBs.

*C*. *burnetii* has been proved to be a potent immunomodulator which can modulate host immune responses positively [23]. Mice injected with phase I *C*. *burnetii* has been showed to increase resistance to tumors and bacteria [19], virus [20] or protozoans [21]. The non-specific immunopreotection by phase I *C*. *burnetii* has been recognized as involving the enhancement of humoral immunoresponses [41] and cellular immunoresponses including the increase of IFN-γ/β level in sera and the percentage of T-cells and la-positive T-cells in spleens [20] as well as macrophage functions [42] which may involve the oxygen radical microbicidal system [23]. High levels of GM-CSF and IL-1 detected after *in vivo* or *in vitro* exposure to phase I *C*. *burnetii* can enhance dendritic cell activity [43]. Furthermore, phase I *C*. *burnetii* can increase expression of la MHC class II antigen to induce production of IFN-γ and other lymphokines in hosts[23]. All these strongly suggest that phase I *C*. *burnetii* is a good immunopotentiator which can stimulate antigen processing cells, leading to enhanced antigen processing and subsequent humoral and cellular immunoresponses.

Meanwhile, phase I *C*. *burnetii* also can negatively modulate host immune responses, inducing immune suppression [44] and adverse reactions giving rise to severe local inflammatory responses such as persistent indurated masses or sterile abscesses at the injection site in immunization [45]. Phase I *C*. *burnetii* lipopolysaccharide is important in the development of immunity [46], but it’s also a major virulence factor containing determinants of immune suppressive and adverse components. Fortunately, the components that can induce the immune suppression and adverse reaction are efficiently dissociated from phase I *C*. *burnetii* by chloroform-methanol extraction [47]. The obtained phase I *C*. *burnetii* CMR has been shown to be nontoxic, immunogenic, and protective in animals and humans [48].

In the present study, rOmpB-4 was chosen to combine with *C*. *burnetii* CMR in immunization of mice so as to explore whether CMR could promote rOmpB-4 to induce more efficient protection against *R*. *rickettsii* infection. Our results showed that mice immunized with rOmpB-4 combined CMR had a significantly lower of rickettsial load or slighter histopathologic lesions in livers, spleens, or lungs compared with mice immunized with rOmpB-4 or CMR alone. The result demonstrated that *C*. *burnetii* CMR could potentiate rOmpB-4 to induce much more efficient protection against *R*. *rickettsii* infection, especially in lungs of mice. Previous studies found that *R*. *rickettsii* organisms primarily infect the microvascular endothelium, leading to systemic spread of the organisms and the major pathophysiological effects, such as increased microvascular permeability and edema in vital organs, the lung and brain [49]. Thus the effective inhibition of rickettsial infection in lung is critical for protection against *R*. *rickettsii* infection.

CMR of *C*. *burnetii* has been well demonstrated to be a safer Q fever vaccine compared with WCV, and vaccination with the CMR can effectively prime the immune system to mount significant anamnestic responses after infection [50]. In the present study, mice immunized with rOmpB-4 combined with *C*. *burnetii* CMR showed that the coxiella load in livers, spleens or lungs was significantly lower than that of mice immunized without CMR. This result suggested that in the combination immunization, *C*. *burnetii* CMR could keep intrinsic immunoprotective efficacy against *C*. *burnetii* infection in addition to be a good immunopotentiator capable to enhance the specific protection conferred by rOmpB-4.

Mice immunized with rOmpB-4 combined with CMR produced a significantly higher level of IgGs to rOmpB-4 and the total amount of rickettsiae in host cells was markedly reduced by sera from the mice in neutralization assay. These results suggested that CMR could efficiently promote humoral responses to rOmpB-4, producing greater amount of specific antibodies to block rickettsia invasion of vascular endothelial cells. Moreover, both IgG1 and IgG2a to rOmpB-4 were markedly in mice immunized with rOmpB-4 combined with CMR, indicating that this combined immunization induced both T helper cell type 2 (Th2) and T helper cell type 1 (Th1) specific immunoresponses [51]. Whereas, the ratio of IgG2a/IgG1 to rOmpB-4 in these mice was markedly higher than that of mice immunized with rOmpB-4 alone during the early phase of vaccination, indicating that CMR could more efficiently increase production of specific IgG2a and the specific immunoresponse was skewing toward Th1 pathway. Previous studies have proved that IgG2a may promote specific immunoprotections and facilitate the removal of intracellular pathogens from hosts. Specifically, the Fc portion of IgG2a can interact with complement components [52] and Fc receptors of macrophages [53] to elicit antibody-dependent cell-mediated cytotoxicity [54] and opsonophagocytosis by macrophages [55], which contributes to clearance of rickettsiae from infected hosts.

In addition, both IFN-γ and TNF-α in mice immunized with rOmpB-4 combined with *C*. *burnetii* CMR were significantly higher than those in mice immunized with rOmpB-4 or CMR alone, indicating that *C*. *burnetii* CMR could effectively facilitate production of both Th1-specific IFN-γ and important inflammatory cytokine TNF-α. IFN-γ and TNF-α produced in Th1-oriented immunoresponses are critical for eradication of rickettsial infection in hosts since they can act synergistically to activate endothelial cells and other cells infected by rickettsiae to kill intracellular rickettsiae via a nitric oxide synthesis-dependent mechanism [56]. Moreover, IFN-γ produced by activated T cells, NK cells, macrophages and/or dendritic cells [57] is a potent activator of macrophages and Th1 immunoresponses [58]. TNF-α is also a potent activator of macrophages and the TNF-α-activated macrophages in turn produce greater amounts of IFN-γ to synergize with TNF-α, mediating more potent killing of intracellular bacteria [59,60].

### Conclusion

In the present study, *C*. *burnetii* CMR could effectively potentiate a protein fragment rOmpB-4 derived from OmpB of *R*. *rickettsii* to elicit markedly enhanced protection against *R*. *rickettsii* infection through increasing production of specific antibodies, particular IgG2a, and cytokines IFN-γ and TNF-α, which acted synergistically to resist *R*. *rickettsii* infection in mice. In addition, *C*. *burnetii* CMR could keep intrinsic efficacy to induce immunoprotection against *C*. *burnetii* infection in the combined immunization in addition to act as a potent immunopotentiator. Therefore, the combination of protective rOmpBs of *R*. *rickettsii* and CMR of *C*. *burnetii* may provide candidates for development of vaccines against both *R*. *rickettsii* and *C*. *burnetii* infection.

## Supporting Information

S1 Table Primer sequences and cleavage sites of *ompB* fragments. (DOCX)

S2 Table Primers and probes of *R. rickettsii ompB*, *R. conorii 23S rRNA*, and C3H/HeN Mouse *actin*. (DOCX)
