# Supplementary material for: Chloroform-Methanol Residue of Coxiella burnetii Markedly Potentiated the Specific Immunoprotection Elicited by a Recombinant Protein Fragment rOmpB-4 Derived from Outer Membrane Protein B of Rickettsia rickettsii in C3H/HeN Mice
Source: PLoS One. 2015 Apr 24;10(4):e0124664. doi: 10.1371/journal.pone.0124664 (PMC4409375; doi:10.1371/journal.pone.0124664)
Supplement: S1 Table — (DOCX) [file pone.0124664.s001.docx]

**S1 Table. Primer sequences and cleavage sites of *ompB* fragments.**

| Primer name | Cleaving enzyme | cleavage sites | Sequence（5'→3'） | Gene length (bp) |
| --- | --- | --- | --- | --- |
| *ompB-1* Forward (F) | BamHI | 34 | GGGGATCCATTTCCGCAGGGTTG | 1068 |
| *ompB-1*Reverse (R) | XhoI | 1101 | GGCTCGAGATTTACTTGCCCAGTTGC | 1068 |
| *ompB-2 F* | BamHI | 925 | GGGGATCCGGTCAACTTACAGCTA | 1215 |
| *ompB-2 R* | XhoI | 2139 | GGCTCGAGAACATTAACCCCTTTA | 1215 |
| *ompB-3 F* | BamHI | 1960 | GGGGATCCGCAGTACAATTCGCTC | 972 |
| *ompB-3 R* | XhoI | 2931 | GGCTCGAGTGTACCAGGGGTATTAGG | 972 |
| *ompB-4 F* | BamHI | 2638 | GGGGATCCAGAGGCATTCCATTCA | 1185 |
| *ompB-4 R* | XhoI | 3822 | GGCTCGAGGTTCTGATCTACACCCG | 1185 |
| *ompB-5 F* | BamHI | 3454 | GGGGATCCGGAAACAATACTTCTATT | 1494 |
| *ompB5 R* | HindIII | 4947 | GGAAGCTTTTTTAGAGTACCTTGATGT | 1494 |
