# Supplementary material for: Chloroform-Methanol Residue of Coxiella burnetii Markedly Potentiated the Specific Immunoprotection Elicited by a Recombinant Protein Fragment rOmpB-4 Derived from Outer Membrane Protein B of Rickettsia rickettsii in C3H/HeN Mice
Source: PLoS One. 2015 Apr 24;10(4):e0124664. doi: 10.1371/journal.pone.0124664 (PMC4409375; doi:10.1371/journal.pone.0124664)
Supplement: S2 Table — (DOCX) [file pone.0124664.s002.docx]

**S2 Table. Primers and probes of *R. rickettsii ompB*, *R. conorii 23S rRNA,* and C3H/HeN Mouse *actin*.**

| Primer or probe name | Sequence(5'→3') |
| --- | --- |
| *R. rickettsii ompB-F* | TGAAGATACTACCTTAGGGTTCATCACTA |
| *R. rickettsii ompB-R* | ACCGGCATTAAGCGTAAGGTT |
| *R. rickettsii ompB* probe | (6-Fam)-TGTTGTTCATAACGCTCAC-(MGB) |
| *C. burnetii 23S rRNA-F* | CGGCTGAATTTAAGCGATTTATTTT |
| *C. burnetii 23S rRNA-R* | CGTAACCACACACGCATCTCA |
| *C. burnetii 23S rRNA* probe | (6-Fam)-CCGAACCCATTGCAA-(MGB) |
| Mouse C3H/HeN *actin-F* | CCTGTATGCCTCTGGTCGTA |
| Mouse C3H/HeN *actin-R* | CCATCTCCTGCTCGAAGTCT |
| Mouse C3H/HeN *actin* probe | (6-Fam)-ACTGTGCCCATCTACGAG-(MGB) |
